# Quercetin Alleviates Chronic Post-Ischemia Pain in Rats and Inhibits NaV1.7 Sodium Channels

**DOI:** 10.3390/molecules31213809

**Published:** 2026-10-22

**Authors:** Xiao Liu, Tao Liu, Weihang Zhao, Weijie Guo, Haoyi Yang, Jia Chen, Zhiming Shan, Song Cai

**Affiliations:** 1 College of Chemistry and Environmental Engineering, Shenzhen University, Shenzhen 518055, China; xxliu@szu.edu.cn; 2 Health Science Center, Shenzhen University, Shenzhen 518055, China; 2210245055@email.szu.edu.cn (T.L.); 2021221114@email.szu.edu.cn (J.C.); 3 Department of Anatomy and Histology, Shenzhen University Medical School, Shenzhen 518055, China; zwhzhaoweihang@126.com; 4 Department of Anesthesiology, Shenzhen People’s Hospital, The First Affiliated Hospital, Southern University of Science and Technology, Shenzhen 518020, China; 2110245025@email.szu.edu.cn; 5 Department of Medical Neuroscience, School of Medicine, Shenzhen People’s Hospital, The First Affiliated Hospital, Southern University of Science and Technology, Shenzhen 518055, China; 2070245042@email.szu.edu.cn

**Keywords:** quercetin, Nav1.7, neuropathic pain, the chronic post-ischemia pain (CPIP)

## Abstract

Complex Regional Pain Syndrome type I (CRPS-I) poses a formidable challenge in pain management due to its debilitating symptoms and a lack of effective, targeted therapies. The chronic post-ischemia pain (CPIP) rodent model, which recapitulates the ischemia–reperfusion injury implicated in CRPS-I, provides a robust platform for exploring novel interventions. While quercetin (Que), a natural flavonoid, is recognized for its anti-inflammatory and antioxidant properties, its specific therapeutic efficacy and molecular targets in CRPS-I remain largely unknown. In this study, we evaluated the therapeutic potential of Que in addressing CRPS-related neuropathic pain using the CPIP model. Behavioral assessments revealed that Que significantly attenuates mechanical allodynia and cold hypersensitivity in CPIP rats. To elucidate the underlying mechanism, we investigated Que’s impact on peripheral nociceptive signaling. Whole-cell patch-clamp recordings demonstrated that Que markedly suppresses tetrodotoxin-sensitive (TTX-S) sodium currents in isolated dorsal root ganglion (DRG) neurons. To pinpoint the exact molecular target, we further evaluated Que in a heterologous expression system, confirming that it selectively inhibits Nav1.7 channel currents in transfected HEK293 cells. In addition to its known anti-inflammatory profile, our in vitro findings reveal that Que selectively modulates the Nav1.7 channel, providing new insights into its pleiotropic pharmacological characteristics. By bridging macroscopic analgesic phenotypes with a microscopic channel-blocking mechanism, this study establishes Que as a promising, safe, non-opioid candidate for CRPS-I translational therapy.

## 1. Introduction

Complex Regional Pain Syndrome (CRPS) is a severely debilitating chronic pain condition characterized by prolonged regional pain, hyperalgesia, and autonomic dysfunction, typically following trauma or localized ischemia. Its precise etiology remains incompletely understood [1]. Clinically, CRPS is classified into two subtypes based on the absence (CRPS-I) or presence (CRPS-II) of overt peripheral nerve injury [2]. Because CRPS-I develops without identifiable nerve damage and possesses a highly complex pathogenesis—often involving sustained neuroinflammation and neuroplasticity—it represents a profound clinical challenge and an area of intense active investigation.

To investigate the underlying mechanisms of CRPS-I, the chronic post-ischemic pain (CPIP) rodent model has been widely adopted. The CPIP model robustly recapitulates the core pathophysiological and phenotypic features of human CRPS-I, including oxidative stress, severe inflammatory responses, and subsequent peripheral neural sensitization [3]. The use of the CPIP rat model provides a highly reliable and reproducible preclinical platform for uncovering novel analgesic strategies targeting CRPS-I [4].

The dorsal root ganglion (DRG) serves as the primary gateway for transmitting nociceptive signals and is pivotal in the genesis and maintenance of neuropathic pain [5]. Within DRG neurons, voltage-gated sodium channels (VGSCs) dictate cellular excitability and the propagation of action potentials [6]. Among these, the Nav1.7 subtype (encoded by the *SCN9A* gene) is predominantly expressed in peripheral nociceptors. Human genetic studies and preclinical models have unequivocally established that Nav1.7 acts as a critical threshold channel for pain transmission [7,8]. Consequently, selectively targeting Nav1.7 has emerged as a highly promising strategy for the development of non-opioid analgesic therapeutics [9].

Quercetin (Que) is a natural flavonoid compound that has received widespread attention due to its anti-inflammatory, antioxidant, and analgesic properties [10]. Extensive behavioral and pharmacological studies indicate that Que can modulate inter-neuronal signal transduction and alleviate various forms of neuropathic pain [11,12]. Furthermore, a growing body of evidence highlights the ability of Que to interact with diverse ion channels, including voltage-gated sodium channels [13]. However, despite this well-documented mechanistic repertoire, its specific efficacy in the ischemic microenvironment of CRPS-I, as well as its direct electrophysiological interaction with the Nav1.7 channel, remains incompletely defined. Therefore, rather than establishing its general analgesic potential, the present study aims to determine whether Que exerts a targeted inhibitory effect on Nav1.7 function within the framework of the CPIP model, thereby establishing a novel mechanistic rationale for its therapeutic application in chronic ischemic pain.

## 2. Results

### 2.1. Quercetin Exerts Antinociceptive Effects in the CPIP-Induced Rat Model of Neuropathic Pain

Quercetin (C_15_H_10_O_7_, CAS:117-39-5, molecular weight 302.24 g/mol) is a natural dietary flavonoid. Previous studies have reported the analgesic efficacy of Quercetin (Que) in various neuropathic pain models, including chronic constriction injury [14], spared nerve injury model [15] and paclitaxel-induced neuropathic pain model [10]. In the present study, we evaluated the antinociceptive activity of Que in a chronic post-ischemia pain (CPIP) model and investigated its underlying mechanisms. The CPIP model was established by applying a tight O-ring around the left hind ankle of rats for 3 h, inducing transient tissue ischemia and hypoxia characterized by local edema and cyanosis. Reperfusion following O-ring removal typically results in a prolonged decrease in the paw withdrawal threshold lasting for nearly a month [3]. Following CPIP modeling, adult male rats exhibited marked mechanical allodynia, cold hypersensitivity, and mechanical hyperalgesia.

**Figure 1:**
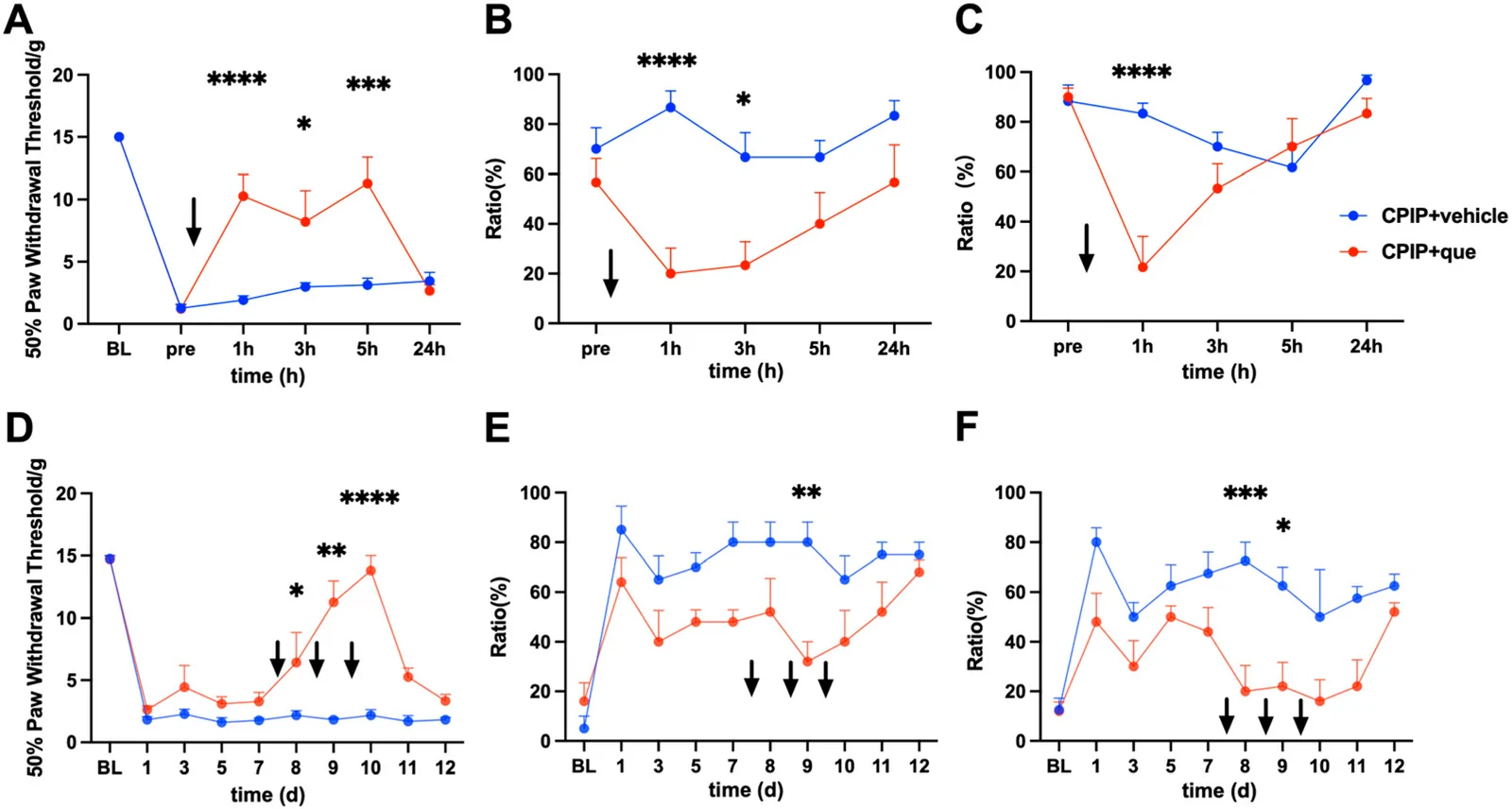
Effects of single and consecutive administrations of Que on pain-related behaviors in the CPIP model. (**A**–**C**) Behavioral responses following a single administration of Que or vehicle. The black downward arrow indicates the time point of the single drug administration. (**A**) Mechanical allodynia assessed by the von Frey test (50% withdrawal threshold). (**B**) Cold hypersensitivity evaluated by acetone noxious withdrawal responses. (**C**) Mechanical hyperalgesia measured by Pin test responses. (**D**–**F**) Behavioral responses following three consecutive administrations of Que or vehicle. The black downward arrows indicate the time points of the repeated drug administrations. (**D**) 50% paw withdrawal threshold in the von Frey test. (**E**) Acetone noxious withdrawal responses. (**F**) Pin test responses. pre: time before i.p.; BL: baseline. Data are presented as mean ± SEM, vehicle *n* = 6, quercetin *n* = 6, * *p* < 0.05, ** *p* < 0.01, *** *p* < 0.001, **** *p* < 0.0001 (Bonferroni’s post hoc test). Two-way repeated measures ANOVA statistics (Que treatment main effect; Time × Treatment interaction) are as follows: (**A**) *F*(1, 59) = 28, *p* < 0.0001; *F*(5, 59) = 7.0, *p* <0.0001. (**B**) *F*(1, 50) = 32.14, *p* < 0.0001; *F*(4, 50) = 2.1, *p* = 0.0868. (**C**) *F*(1, 50) = 10.77, *p* = 0.0019; *F*(4, 50) = 6.0, *p* = 0.0005. (**D**) *F*(1, 70) = 65, *p* < 0.0001; *F*(9, 70) = 7.4, *p* < 0.0001. (**E**) *F*(1, 70) = 29, *p* < 0.0001; *F*(9, 70) = 1.4, *p* = 0.1873. (**F**) *F*(1, 70) = 42, *p* < 0.0001; *F*(9, 70) = 1.5, *p* < 0.1486.

To assess the acute effects of Que, rats received a single intraperitoneal (i.p.) injection of either vehicle (10% DMSO, 10% Tween-80, and 80% saline) or Que (100 mg/kg). Behavioral testing revealed that a single administration of Que significantly reversed CPIP-induced mechanical allodynia, with the 50% withdrawal threshold peaking at 1 to 5 h post-injection (Figure 1A; Two-way ANOVA, treatment main effect: *F*(1, 59) = 28.0, *p* < 0.0001; time × treatment interaction: *F*(5, 59) = 7.0, *p* < 0.0001). Similarly, cold hypersensitivity and mechanical hyperalgesia were significantly, albeit transiently, attenuated following the single Que administration (Figure 1B,C; Acetone test treatment main effect: *F*(1, 50) = 32.14, *p* < 0.0001, interaction: *F*(4, 50) = 2.1, *p* = 0.0868; Pin test treatment main effect: *F*(1, 50) = 10.77, *p* = 0.0019, interaction: *F*(4, 50) = 6.0, *p* = 0.0005).

To further determine the effects of consecutive treatments, CPIP rats were administered Que or vehicle for three consecutive days (days 7, 8, and 9 post-surgery). Repeated Que treatment produced a robust and cumulative increase in the mechanical withdrawal threshold (Figure 1D; treatment main effect: *F*(1, 70) = 65.0, *p* < 0.0001; time × treatment interaction: *F*(9, 70) = 7.4, *p* < 0.0001). Furthermore, cold allodynia and mechanical hyperalgesia were also notably ameliorated during the dosing period, although the behavioral responses exhibited some fluctuation (Figure 1E,F; Acetone test treatment main effect: *F*(1, 70) = 29.0, *p* < 0.0001, interaction: *F*(9, 70) = 1.4, *p* = 0.1873; Pin test treatment main effect: *F*(1, 70) = 42.0, *p* < 0.0001, interaction: *F*(9, 70) = 1.5, *p* = 0.1486). Collectively, these proof-of-concept findings provide compelling initial evidence that a 100 mg/kg systemic dose of Que effectively alleviates ischemia–reperfusion injury-induced neuropathic pain, underscoring the need for future detailed dose–response evaluations.

### 2.2. Quercetin Reduces Total Sodium Current in Small-Diameter DRG Sensory Neurons

**Figure 2:**
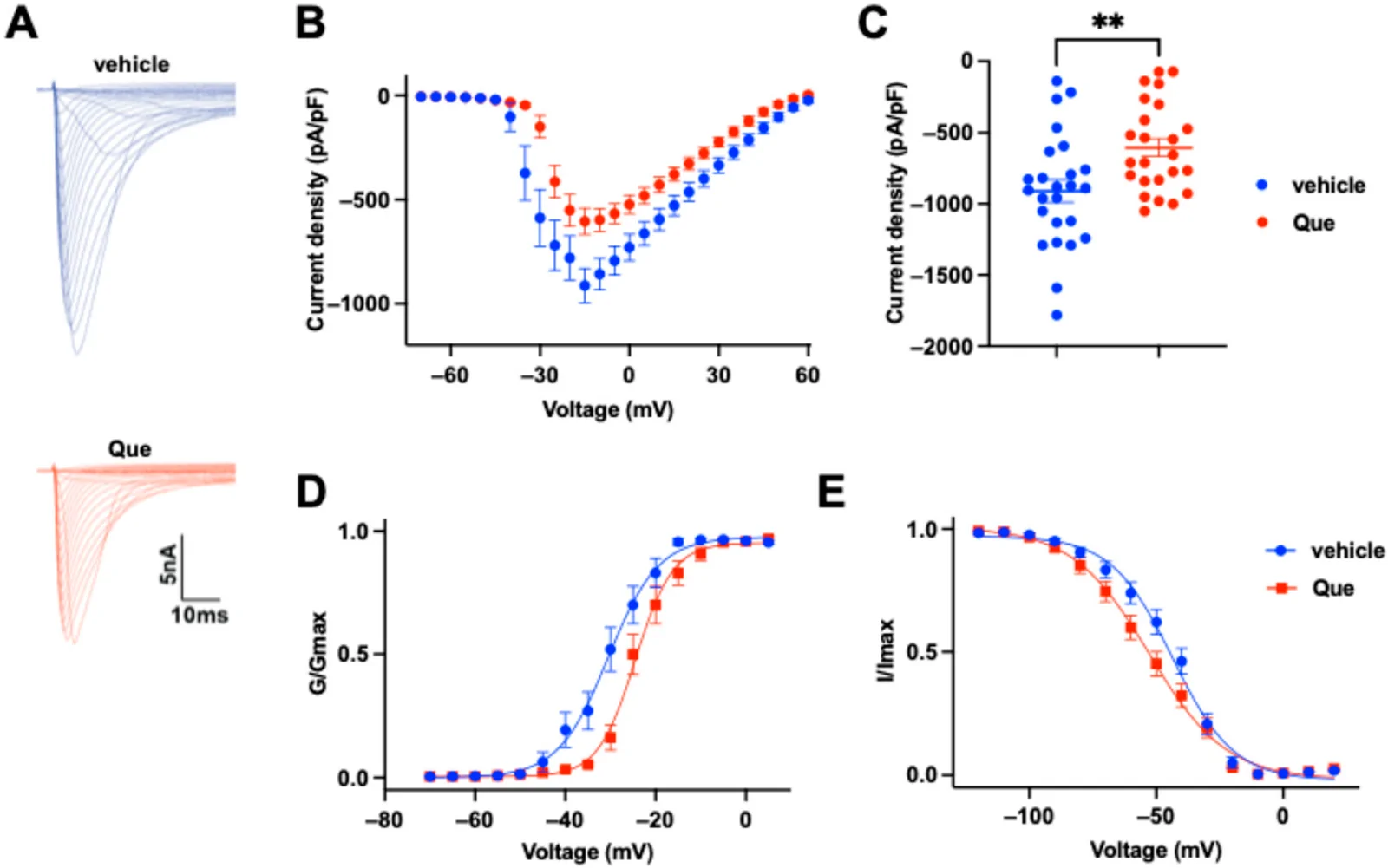
Quercetin inhibits total sodium currents in DRG sensory neurons. (**A**) Representative traces of total NaV currents from DRG sensory neurons treated with 0.1% DMSO control or 20 µM Que. (**B**) Current–voltage (I-V) relationship curves of NaV current density in the vehicle and Que group. (**C**) Scatter plot comparing the peak NaV current density between the vehicle and Que groups. (**D**) Steady-state activation curves fitted with the Boltzmann equation. Normalized conductance (G/Gmax) is plotted as a function of test potentials. (**E**) Steady-state inactivation curves fitted with the Boltzmann equation. Normalized current (I/Imax) is plotted as a function of prepulse potentials. The half-maximal voltages (V_1/2_) for activation and inactivation are summarized in Table 1. *n* = 25 neurons in the control group, *n* = 24 neurons in the Que group. Data are expressed as means ± SEM. ** *p* < 0.01. Statistical analysis for peak current density (**C**) was performed using an unpaired two-tailed *t*-test with Welch’s correction: *t*(45.13) = 3.011, *p* = 0.0043.

To explore the underlying mechanism, we examined the effect of Que on voltage-gated sodium (Nav) currents in small-diameter (<30 µm) DRG neurons using whole-cell patch-clamp recording. Representative families of total NaV current traces recorded from neurons treated overnight with vehicle (0.1% DMSO) or 20 µM Que are shown in Figure 2A. The current–voltage (I-V) relationship analysis revealed that Que treatment significantly inhibited the total NaV currents across a wide range of test potentials (Figure 2B). Specifically, Que reduced the peak sodium current density by approximately 31.9% compared to the control group (vehicle: −909.00 ± 79.41 pA/pF vs. Que: −603.41 ± 63.18 pA/pF; *t*(45.13) = 3.011, *p* = 0.0043, Welch’s *t*-test; Figure 2C). Furthermore, we assessed the effect of Que on the gating kinetics of NaV channels. Que induced a significant depolarizing (rightward) shift in the steady-state activation curve (Figure 2D; V_1/2_, vehicle: −29.03 ± 0.64 mV vs. Que: −24.46 ± 0.49 mV, *p* < 0.0001). Conversely, the steady-state inactivation curve exhibited a significant hyperpolarizing (leftward) shift in the presence of Que (Figure 2E; V_1/2_, vehicle: −41.55 ± 1.06 mV vs. Que: −52.86 ± 1.30 mV, *p* < 0.0001). Taken together, these results support the hypothesis that Que alleviates neuropathic pain in part by suppressing the excitability of DRG sensory neurons through the inhibition of NaV channel activity.

**Table 1:** Effects of quercetin on the gating kinetics of voltage-gated sodium channels in DRG neurons and HEK293 cells.

			0.1% DMSO	Quercetin 20 μM	Statistics (*t*, *df*)	*p* Value
Total NaV	Activation	V_1/2_	−29.03 ± 0.64	−24.46 ± 0.49	t = 5.614, *df* = 47	**** *p* < 0.0001
		k	3.56 ± 0.56	4.16 ± 0.43	*t* = 0.8494, *df* = 47	*p* = 0.4000
	Inactivation	V_1/2_	−41.55 ± 1.06	−52.86 ± 1.30	*t* = 5.823, *df* = 35	**** *p* < 0.0001
		k	−10.38 ± 0.95	−15.23 ± 1.27	*t* = 2.599, *df* = 35	* *p* = 0.0136
Blocked TTX-S	Activation	V_1/2_	−27.03 ± 1.25	−19.18 ± 0.81	*t* = 4.886, *df* = 26	**** *p* < 0.0001
		k	8.00 ± 1.19	5.00 ± 0.70	*t* = 1.984, *df* = 26	*p* = 0.0579
	Inactivation	V_1/2_	−31.98 ± 0.37	−25.91 ± 0.59	*t* = 9.127, *df* = 26	**** *p* < 0.0001
		k	5.00 ± 0.32	6.00 ± 0.51	*t* = 1.738, *df* = 26	*p* = 0.0941
Blocked NaV1.7	Activation	V_1/2_	−26.01 ± 0.69	−22.60 ± 0.42	*t* = 4.010, *df* = 29	*** *p* = 0.0004
		k	4.00 ± 0.61	3.00 ± 0.36	*t* = 1.342, *df* = 29	*p* = 0.1900
	Inactivation	V_1/2_	−30.98 ± 1.09	−43.79 ± 1.62	*t* = 6.762, *df* = 29	**** *p* < 0.0001
		k	9.00 ± 0.96	14.00 ± 1.56	*t* = 2.839, *df* = 29	** *p* = 0.0082
Blocked Nav1.6	Activation	V_1/2_	−25.17 ± 0.59	−23.23 ± 0.57	*t* = 3.706, *df* = 30	*** *p* = 0.0009
		k	4.00 ± 0.53	4.00 ± 0.50	*t* = 0.000, *df* = 30	*p* > 0.9999
	Inactivation	V_1/2_	−30.98 ± 1.07	−43.42 ± 1.85	*t* = 5.819, *df* = 30	**** *p* < 0.0001
		k	9.00 ± 0.95	15.00 ± 1.79	*t* = 2.961, *df* = 30	** *p* = 0.0060
HEK293 NaV1.7	Activation	V_1/2_	−22.11 ± 0.81	−12.21 ± 0.98	*t* = 7.851, *df* = 28	**** *p* < 0.0001
		k	5.00 ± 0.72	6.00 ± 0.74	*t* = 0.9660, *df* = 28	*p* = 0.3423
	Inactivation	V_1/2_	−59.95 ± 0.38	−57.96 ± 0.64	*t* = 2.768, *df* = 28	** *p* = 0.0099
		k	7.00 ± 0.33	8.00 ± 0.56	*t* = 1.581, *df* = 28	*p* = 0.1252

All electrophysiological parameters presented in Table 1 were acquired from native primary DRG neurons, with the exception of the final section (HEK293 Nav1.7), which was recorded from transfected HEK293 cells. Values are means ± SEM calculated from fits of the data from the indicated number of individual cells to the Boltzmann equation; V_1/2_, midpoint potential (mV) for voltage-dependent activation or inactivation; k, slope factor; Significantly different from the value for DMSO (* *p* < 0.05, ** *p* < 0.01, *** *p* < 0.001, **** *p* < 0.0001, unpaired Student’s *t*-test).

### 2.3. Quercetin Preferentially Inhibits Nav1.7 Among TTX-Sensitive Sodium Channels in DRG Sensory Neurons

**Figure 3:**
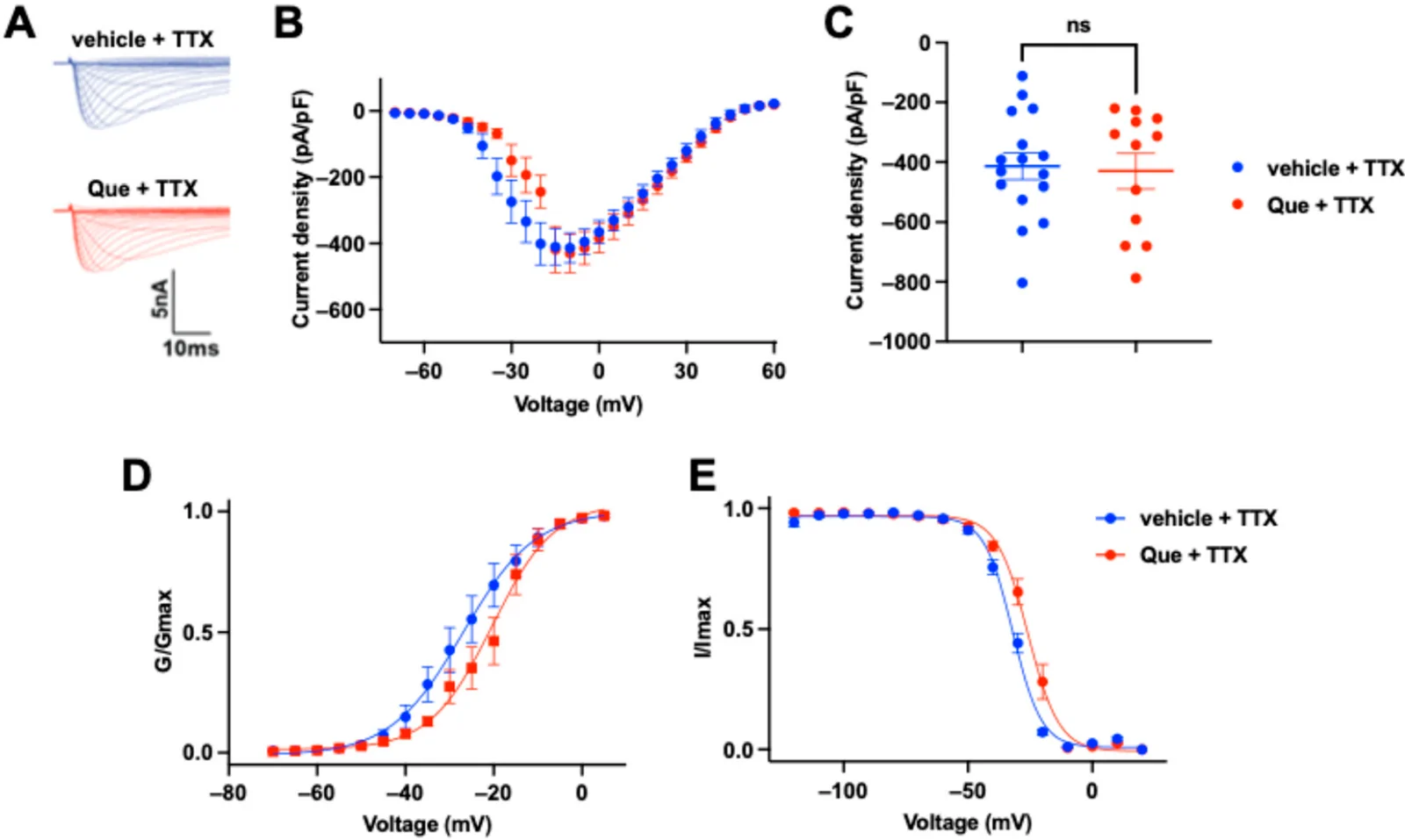
Effects of quercetin on tetrodotoxin-resistant voltage-gated sodium currents in DRG neurons. (**A**) Representative traces of tetrodotoxin-resistant (TTX-R) NaV currents recorded from DRG sensory neurons treated with vehicle (0.1% DMSO) or 20 µM Que. All extracellular solutions contained 1 µM TTX to isolate TTX-R currents. (**B**) Current–voltage (I-V) relationships of the TTX-R sodium current density for the indicated groups. (**C**) Scatter plot summarizing the peak TTX-R NaV current density. (**D**) Steady-state activation and (**E**) steady-state inactivation curves. Normalized conductance (G/Gmax) and normalized current (I/Imax) are plotted as functions of voltage. Solid lines represent fits to the Boltzmann equation. The half-maximal voltages (V_1/2_) for activation and inactivation are summarized in Table 1. Data are expressed as mean ± SEM (*n* = 16 neurons in the vehicle + TTX group, *n* = 12 neurons in the Que + TTX group). ns, not significant. Statistical analysis for peak current density (**C**) was performed using an unpaired two-tailed *t*-test with Welch’s correction: *t*(21.95) = 0.2134, *p* = 0.8330.

Voltage-gated sodium (NaV) channels in DRG neurons are broadly classified into tetrodotoxin-sensitive (TTX-S; Nav1.1, 1.3, 1.6, and 1.7) and TTX-resistant (TTX-R; Nav1.8 and 1.9) subtypes. To determine whether Que acts as a selective inhibitor for specific NaV channel subtypes, we first isolated TTX-R currents by treating neurons with 1 µM TTX. Que failed to significantly reduce the peak current density of TTX-R currents (vehicle + TTX: −414.0 ± 44.82 pA/pF vs. Que + TTX: −429.83 ± 59.14 pA/pF; *t*(21.95) = 0.2134, *p* = 0.8330, Welch’s *t*-test; Figure 3A–C), indicating that its inhibitory effects are predominantly restricted to TTX-S channels. Interestingly, Que altered the gating kinetics of TTX-R channels, causing a depolarizing (rightward) shift in both the steady-state activation (Figure 3D; V_1/2_, vehicle + TTX: −27.03 ± 1.25 mV vs. Que + TTX: −19.18 ± 0.81 mV; *p* < 0.0001) and inactivation curves (Figure 3E; V_1/2_, vehicle + TTX: −31.98 ± 0.37 mV vs. Que + TTX: −25.91 ± 0.59 mV; *p* < 0.0001).

**Figure 4:**
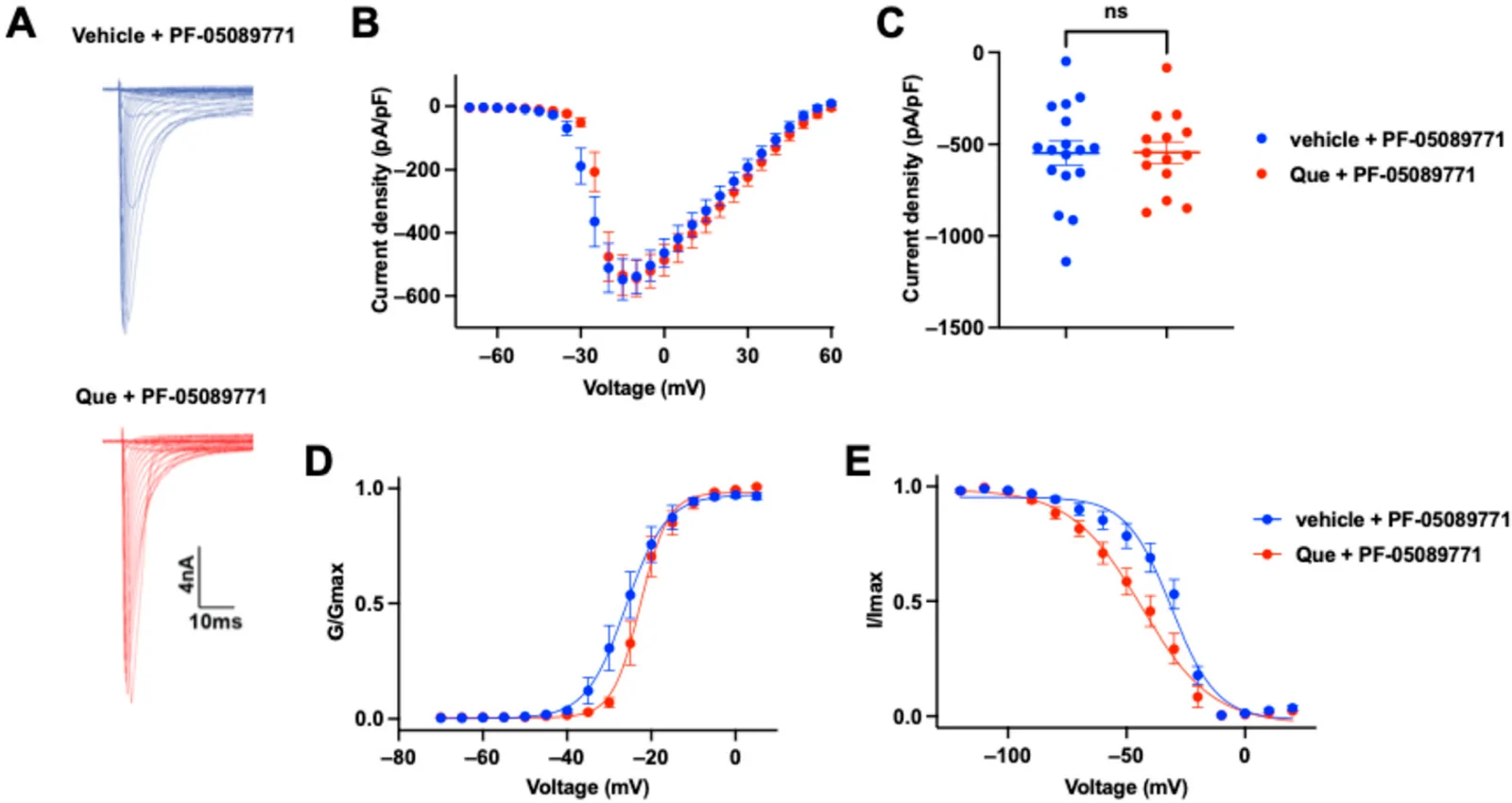
The Nav1.7 inhibitor PF-05089771 abolishes the inhibitory effect of quercetin on sodium currents in DRG sensory neurons. (**A**) Representative traces of voltage-gated sodium currents recorded from DRG sensory neurons treated with vehicle + 100 nM PF-05089771 or 20 µM Que + 100 nM PF-05089771. (**B**) Current–voltage (I-V) relationships of sodium current density for the indicated groups. (**C**) Scatter plot summarizing the peak sodium current density. (**D**) Steady-state activation and (**E**) steady-state inactivation curves. Normalized conductance (G/Gmax) and normalized current (I/Imax) are plotted as functions of voltage. Solid lines represent fits to the Boltzmann equation. The half-maximal voltages (V_1/2_) for activation and inactivation are summarized in Table 1. Data are expressed as mean ± SEM (*n* = 17 neurons in the vehicle + PF-05089771 group, *n* = 14 neurons in the Que + PF-05089771 group). ns, not significant. Statistical analysis for peak current density (**C**) was performed using an unpaired two-tailed *t*-test with Welch’s correction: *t*(29.00) = 0.03215, *p* = 0.9746.

**Figure 5:**
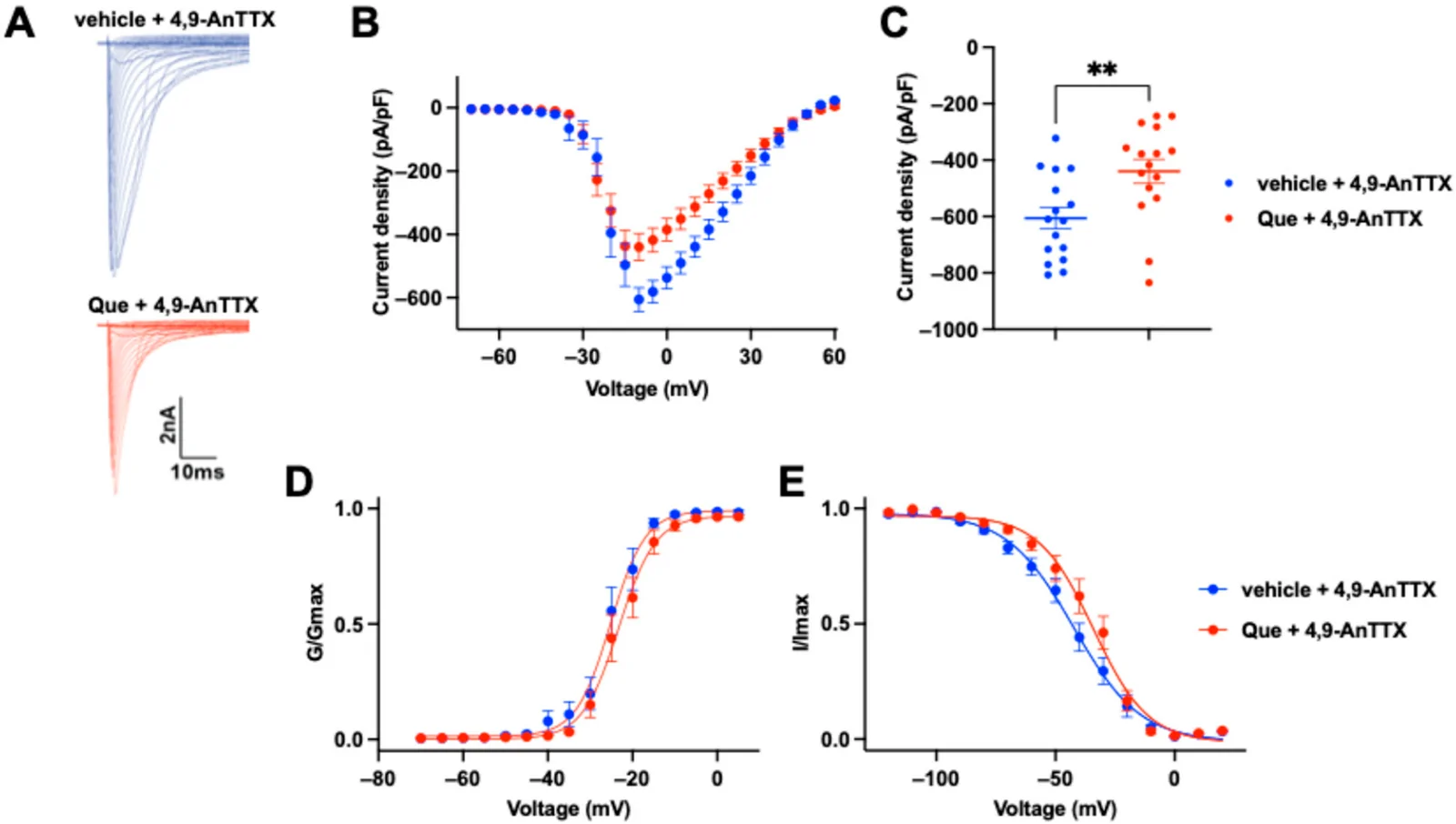
The Nav1.6 inhibitor 4,9-AnTTX does not blunt the inhibitory effect of quercetin on sodium currents in DRG sensory neurons. (**A**) Representative traces of voltage-gated sodium currents recorded from DRG sensory neurons treated with vehicle (0.1% DMSO) + 30 nM 4,9-AnTTX or 20 µM Que + 30 nM 4,9-AnTTX. (**B**) Current–voltage (I-V) relationships of sodium current density for the indicated groups. (**C**) Scatter plot summarizing the peak sodium current density. (**D**) Steady-state activation and (**E**) steady-state inactivation curves. Normalized conductance (G/Gmax) and normalized current (I/Imax) are plotted as functions of voltage. Solid lines represent fits to the Boltzmann equation. The half-maximal voltages (V_1/2_) for activation and inactivation are summarized in Table 1. Data are expressed as mean ± SEM (*n* = 16 neurons in the vehicle + 4,9-AnTTX group, *n* = 16 neurons in the Que + 4,9-AnTTX group). ** *p* < 0.01 compared with the vehicle + 4,9-AnTTX group. Statistical analysis for peak current density (**C**) was performed using an unpaired two-tailed *t*-test with Welch’s correction: *t*(29.56) = 2.922, *p* = 0.0066.

It should be noted that native DRG neurons express a highly heterogeneous population of NaV channel subtypes. Consequently, the application of selective pharmacological blockers (e.g., TTX, PF-05089771, or 4,9-AnTTX) to isolate specific currents inherently shifts the baseline steady-state activation and inactivation kinetics compared to the total unblocked NaV current, as entirely different channel ensembles are being recorded. To further pinpoint the specific TTX-S isoform targeted by Que, we co-administered Que with the highly selective Nav1.7 blocker PF-05089771 (100 nM). In the presence of PF-05089771, the inhibitory effect of Que on the peak sodium current density was completely abolished (vehicle + PF-05089771: −547.4 ± 64.89 pA/pF vs. Que + PF-05089771: −544.58 ± 57.72 pA/pF; *t*(29.00) = 0.03215, *p* = 0.9746, Welch’s *t*-test; Figure 4A–C). This occlusion strongly suggests that Nav1.7 is the primary target for Que’s inhibitory action. In this condition, Que treatment resulted in a depolarizing shift in activation (Figure 4D; V_1/2_, vehicle + PF-05089771: −26.01 ± 0.69 mV vs. Que + PF-05089771: −22.60 ± 0.42 mV; *p* < 0.001) and a hyperpolarizing (leftward) shift in inactivation (Figure 4E; V_1/2_, vehicle + PF-05089771: −30.98 ± 1.09 mV vs. Que + PF-05089771: −43.79 ± 1.62 mV; *p* < 0.0001). Conversely, when currents were recorded in the presence of the selective Nav1.6 blocker 4,9-AnTTX (30 nM), Que retained its ability to significantly inhibit the peak sodium current density (vehicle + 4,9-AnTTX: −605.9 ± 37.75 pA/pF vs. Que + 4,9-AnTTX: −439.4 ± 42.7 pA/pF; *t*(29.56) = 2.922, *p* = 0.0066, Welch’s *t*-test; Figure 5A–C). This demonstrates that blocking Nav1.6 does not prevent Que from exerting its effect. Under these conditions, Que treatment induced a depolarizing (rightward) shift in both the steady-state activation (Figure 5D; V_1/2_, vehicle + 4,9-AnTTX: −25.17 ± 0.59 mV vs. Que + 4,9-AnTTX: −23.23 ± 0.57 mV; *p* < 0.01) and inactivation curves (Figure 5E; V_1/2_, vehicle + 4,9-AnTTX: −30.98 ± 1.07 mV vs. Que + 4,9-AnTTX: −43.42 ± 1.85 mV; *p* < 0.0001). Collectively, these pharmacological occlusion experiments provide compelling evidence that Que preferentially inhibits the TTX-S Nav1.7 channel to exert its effects.

### 2.4. Quercetin Directly Inhibits Recombinant Nav1.7 Channels Expressed in HEK293 Cells

**Figure 6:**
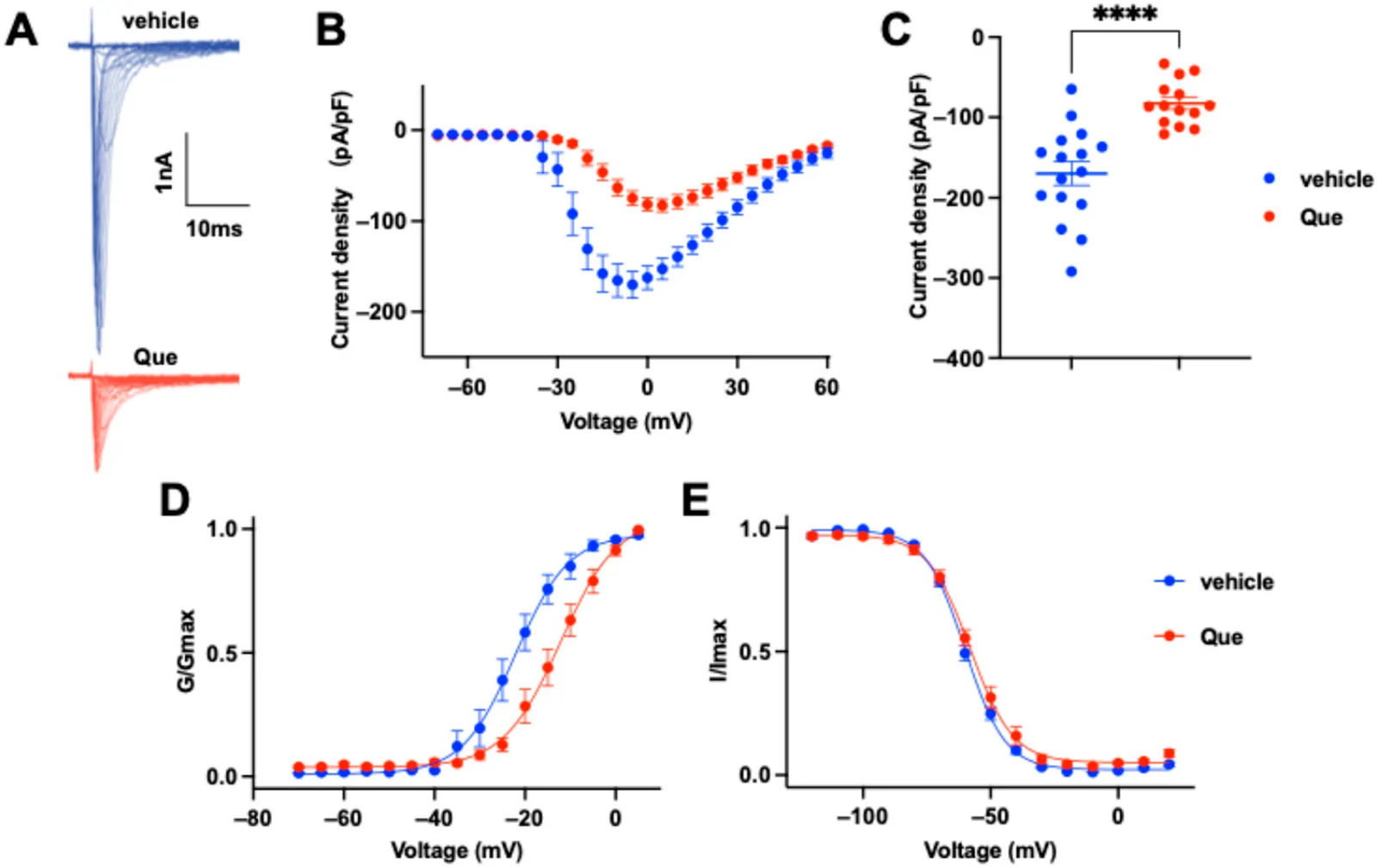
Quercetin specifically inhibits Nav1.7 currents in HEK293 cells. (**A**) Representative traces of Nav1.7 currents recorded from HEK293 cells treated with vehicle (0.1% DMSO) or 20 µM Que. (**B**) Current–voltage (I-V) relationships of Nav1.7 current density for the indicated groups. (**C**) Scatter plot summarizing the peak Nav1.7 current density. (**D**) Steady-state activation and (**E**) steady-state inactivation curves. Normalized conductance (G/Gmax) and normalized current (I/Imax) are plotted as functions of voltage. Solid lines represent fits to the Boltzmann equation. The half-maximal voltages (V_1/2_) for activation and inactivation are summarized in Table 1. Data are expressed as mean ± SEM (*n* = 16 cells in the vehicle group, *n* = 14 cells in the Que group). **** *p* < 0.0001 compared with the vehicle group. Statistical analysis for peak current density (**C**) was performed using an unpaired two-tailed *t*-test with Welch’s correction: *t*(21.91) = 5.294, *p* < 0.0001.

To definitively confirm that Nav1.7 is a direct molecular target of Que, we evaluated its effects on heterologously expressed Nav1.7 channels. HEK293 cells were transiently co-transfected with a pcDNA3.1-Nav1.7-Flag plasmid and a GFP reporter plasmid. Following an overnight incubation with either vehicle (0.1% DMSO) or 20 μM Que, whole-cell patch-clamp recordings were performed. Consistent with our findings in primary DRG neurons, Que markedly suppressed the recombinant Nav1.7 currents in this expression system (Figure 6A,B). Specifically, Que treatment resulted in an approximately 54% reduction in the peak Nav1.7 current density compared to the vehicle control (Vehicle: −170.1 ± 14.78 pA/pF vs. Que: −82.5 ± 7.43 pA/pF; *t*(21.91) = 5.294, *p* < 0.0001, Welch’s *t*-test; Figure 6C). Furthermore, analysis of the gating kinetics revealed that Que significantly altered the voltage-dependent properties of the recombinant channels. As illustrated in Figure 6D,E, Que induced a pronounced depolarizing (rightward) shift in both the steady-state activation and inactivation curves (detailed V_1/2_ values are summarized in Table 1). Taken together, these data provide direct and compelling evidence that Que acts as a potent inhibitor of the Nav1.7 channel.

### 2.5. Computational Studies Supporting Quercetin Binding

**Figure 7:**
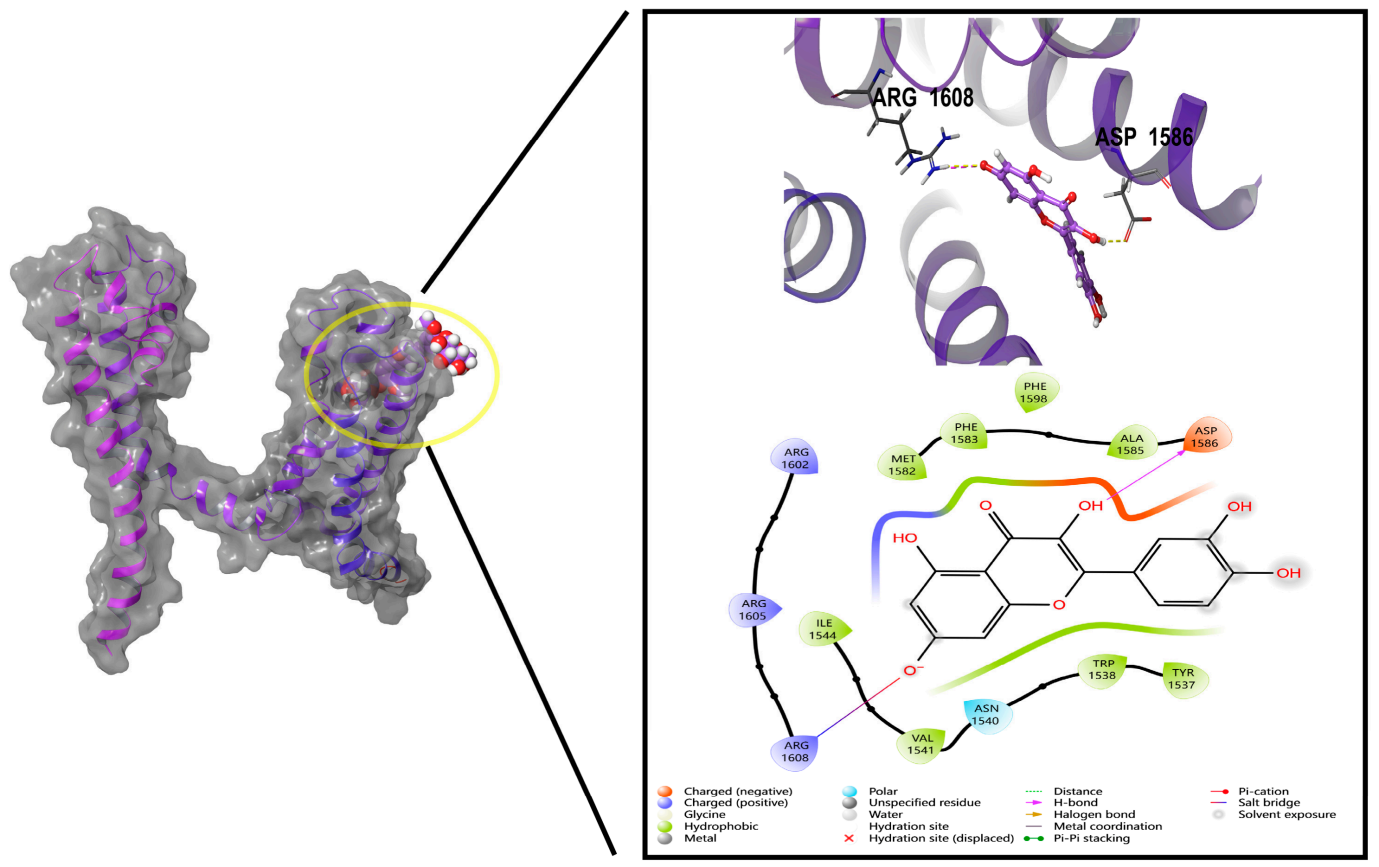
Molecular docking model of the quercetin–Nav1.7 complex. (**Left**) Global overview of the Nav1.7 channel (PDB: 5EK0) showing the binding pose of Que (depicted in sphere representation) within the putative pocket. (**Top right**) Close-up 3D view of the binding site, highlighting key interactions between Que and surrounding residues (ARG1608 and ASP1586), with hydrogen bonds and salt bridges represented by yellow dashed lines. (**Bottom right**) Two-dimensional (2D) interaction diagram detailing the comprehensive network of residue contacts, including hydrogen bonds, salt bridges, and hydrophobic interactions between Que and the Nav1.7 pocket.

Given that Que reduced total voltage-gated Na^+^ currents while leaving the TTX-resistant component largely unchanged, we next investigated Nav1.7 as a candidate TTX-sensitive molecular target. A molecular docking experiment was conducted between Que and Nav1.7. The predicted binding pose positioned Que within the extracellular VSD4 ligand-binding pocket and revealed two principal polar anchoring interactions. In the ionization state used for docking, the deprotonated 7-oxygen on the A ring formed a salt bridge with the guanidinium group of Arg1608, whereas the 3-hydroxyl group on the heterocyclic C ring projected toward and formed a hydrogen bond with the side-chain carboxylate of Asp1586 (Figure 7). The planar flavonol scaffold was further accommodated by a predominantly hydrophobic microenvironment lined by Tyr1537, Trp1538, Val1541, Ile1544, Met1582, Phe1583, Ala1585, and Phe1598, with several charged or polar residues, including Asn1540, Arg1602, and Arg1605, positioned around the pocket. Collectively, these predicted electrostatic, hydrogen-bonding, and hydrophobic interactions suggest favorable accommodation of Que within the Nav1.7 VSD4 pocket and provide a plausible structural explanation for the suppression of total Na^+^ currents in the absence of a significant effect on the TTX-resistant component.

## 3. Discussion

In the present study, extending multiple earlier published reports showing the efficacy of quercetin (Que) against various forms of neuropathic pain, we demonstrated that Que effectively alleviates mechanical allodynia, cold hypersensitivity, and mechanical hyperalgesia in the chronic post-ischaemic pain (CPIP) model, a well-established rodent model of complex regional pain syndrome type I (CRPS I). More importantly, electrophysiological evaluations revealed that Que preferentially suppresses tetrodotoxin-sensitive (TTX-S) sodium currents, specifically targeting Nav1.7 channels, which is further supported by molecular docking simulations.

Quercetin is a well-characterized natural flavonoid with potent antioxidant, anti-inflammatory, and analgesic properties [16,17]. Its anti-allodynic efficacy has been robustly documented in various pain conditions, such as diabetic neuropathy, paclitaxel-induced neuropathy, and chronic constriction injury models [10,12,14]. Building upon this extensive behavioral foundation, the present study was not designed to claim the first discovery of Que’s general analgesia. Instead, it serves as an electrophysiological extension that explicitly adds Nav1.7 modulation to the compound’s well-documented mechanistic repertoire. By validating its behavioral efficacy in the previously unexplored CPIP model, we provide compelling evidence that systemic administration of Que significantly reverses ischemia–reperfusion-induced hypersensitivity, mechanistically bridged by its ability to alter Nav1.7 gating dynamics.

Voltage-gated sodium (NaV) channels are critical regulators of peripheral neuronal excitability and represent validated targets for therapeutic intervention in pain management [18]. Due to the diverse expression profiles of NaV subtypes, establishing drug selectivity is paramount. Our subtype-specific pharmacological isolation demonstrated that Que’s inhibitory action bypasses TTX-resistant channels and Nav1.6, steering the focus directly toward Nav1.7. This was further substantiated by our findings in heterologously expressed Nav1.7 channels in HEK293 cells, where sustained exposure to Que markedly suppressed peak current densities and altered gating kinetics. Furthermore, while Que induced an approximately 32% reduction in peak current density in native DRG neurons, a more robust inhibition (~54%) was observed in transfected HEK293 cells. This discrepancy likely stems from the nature of the heterologous expression system. In HEK293 cells, Nav1.7 is transiently overexpressed at high densities under a strong promoter, lacking the competitive presence of auxiliary β-subunits, scaffolding proteins, and other Nav isoforms (e.g., Nav1.8/1.9) that natively co-exist and modulate drug-channel interactions in primary DRG neurons. Combined with our molecular docking models—showing stable binding conformations mediated by key residues such as ARG1608 and ASP1586—these results confirm Nav1.7 as a direct functional target of Que.

Beyond direct channel modulation, the therapeutic profile of Que is deeply intertwined with its antioxidant and anti-inflammatory attributes. CPIP pathology is characterized by marked lower limb edema, cyanosis, and elevated local inflammatory mediators, including myeloperoxidase, pentraxin 3, and complement components [19], which heighten nociceptor sensitivity. Concurrently, ischemia–reperfusion injury triggers enhanced vesicle transport and augmented current amplitudes in DRG neurons [20]. The flavonoid core structure of Que enables efficient hydrogen-atom donation to neutralize free radicals, stabilize conjugated systems, and chelate metal ions to suppress Fenton reactions [21]. Whether these multi-target antioxidant actions synergize with Nav1.7 conformational shifts to modulate channel trafficking and membrane availability warrants future investigation.

Despite these robust findings, several limitations of our study must be explicitly acknowledged. First, while our electrophysiological data reveal a novel interaction between Que and Nav1.7 gating, we recognize that attributing the robust in vivo analgesia solely to Nav1.7 inhibition is premature. Prior studies have established Que’s pleiotropic nature, showing it can modulate alternative targets regulating peripheral nociception, including ASIC channels [22], hERG potassium channels [23], 5-HT3 receptors [24], α7 nicotinic acetylcholine receptors [25] and L-type calcium channels [26]. Furthermore, pharmacokinetic studies indicate that following systemic administration, the concentration of free, unconjugated Que in relevant tissues (e.g., spinal cord) may be limited due to rapid metabolism into glucuronide and sulfate conjugates [27]. Importantly, recent evidence highlights that selective inhibition of Nav1.7 channels at the central terminals of primary sensory neurons within the spinal cord is a key determinant for eliciting in vivo analgesia [28]. For context, clinical-stage selective Nav1.7 inhibitors, such as MK-2075, require approximately 50% target inhibition to produce acute efficacy [29]. Given that a 20 µM concentration did not achieve half-maximal inhibition in our in vitro assays, the in vivo antinociceptive effects observed at the 100 mg/kg dose are likely driven by a synergistic mechanism, where Que’s antioxidant and anti-inflammatory properties act in concert with its modulatory effect on peripheral sodium channels. To address this mechanistic complexity, future studies utilizing a panel of additive pharmacological inhibitors (e.g., combining Nav1.7 blockers with L-type calcium channel antagonists) are strictly required to dissect the exact proportional contributions of these diverse targets to the overall in vivo analgesia. Second, relying on a single in vivo dose and a single in vitro concentration is a distinct limitation of this proof-of-concept study, precluding the calculation of a precise IC50, therapeutic window, or in vivo target engagement dynamics. Therefore, to prevent overinterpretation, future comprehensive dose–response evaluations coupled with detailed pharmacokinetic profiling are strictly required. Third, regarding the behavioral fluctuations observed during the consecutive administration paradigm, while we hypothesize that this transient attenuation may reflect the dynamic and rapidly escalating neuroinflammatory cascade intrinsic to the ischemia–reperfusion injury, we explicitly acknowledge that without direct antinociceptive tolerance assays, this explanation remains speculative. Future investigations must incorporate direct tolerance testing to definitively rule out classical receptor desensitization. Finally, to ensure broader translational validity, future investigations must fully elucidate the downstream intracellular signaling cascades triggered by Que binding, include female subjects to address potential sex-specific differences, and ultimately involve clinical trials to determine whether these therapeutic benefits translate to human patients.

## 4. Materials and Methods

### 4.1. Animals

Adult male Sprague Dawley (SD) rats, procured from the Guangdong Experimental Animal Center (Guangzhou, China), were utilized in this study. The rats were maintained in a specific pathogen-free facility with ad libitum access to standard chow and water. Housing conditions included a regulated 12-h light/dark illumination cycle and a stable ambient temperature of 23 ± 3 °C. The experimental protocols received ethical approval from the Institutional Animal Care and Use Committee at Shenzhen University Medical School. All in vivo interventions strictly adhered to the ethical principles mandated by the International Association for the Study of Pain (IASP) and local laboratory animal care guidelines.

### 4.2. Reagents

Standard chemical reagents were procured from Sigma-Aldrich (St. Louis, MO, USA) unless stated otherwise. Cell culture media and supplements, including DMEM (Cat. No. 2318815), fetal bovine serum (Cat. No. 2176404), and penicillin/streptomycin (Cat. No. 2321127), were supplied by Gibco (Thermo Fisher Scientific, Waltham, MA, USA). Enzymes for tissue digestion, specifically neutral protease (Cat. No. LS02104) and type I collagenase (Cat. No. LS004194), were sourced from Worthington (Lakewood, NJ, USA). Poly-D-lysine hydrobromide was provided by Sigma (Cat. No. P7886) (St. Louis, MO, USA), and the JetPrime transfection kit was acquired from Polyplus (Cat. No. 101000046) (Illkirch, France). Pharmacological agents and vehicles, including Quercetin (Que, HY-18085), PF-05089771 (HY-12883), 4,9-anhydrotetrodotoxin (4,9-AnTTX, HY-126985), PEG300 (HY-Y0873), Tween-80 (HY-Y1891), and dimethyl sulfoxide (DMSO, HY-Y0320), were all purchased from MedChemExpress (MCE) (Monmouth Junction, NJ, USA). Tetrodotoxin (TTX) was acquired from Alomone Labs (Jerusalem, Israel).

### 4.3. Hindpaw Ischemia and Reperfusion

To induce the chronic post-ischemia pain (CPIP) model, we adopted a well-established tourniquet methodology [3]. Animals were maintained under deep anesthesia for approximately 3 h using an intraperitoneal injection of 2% pentobarbital sodium (induction: 40 mg/kg; maintenance: 20 mg/kg). Briefly, a 7/32-inch Nitrile 70 Durometer O-ring was positioned just proximal to the left ankle joint using a modified 1.5-mL microcentrifuge tube as a guide. The guide tube was subsequently removed, allowing the O-ring to constrict the limb and trigger regional ischemia. Exactly 3 h later, the constricting O-ring was severed to initiate reperfusion. Control animals (sham group) were subjected to the identical anesthetic duration but did not undergo hindlimb tourniquet application.

### 4.4. Von Frey Test

Tactile allodynia was quantified via the von Frey filament assay. Prior to measurement, each rat was habituated for a minimum of 30 min in a transparent acrylic enclosure resting on an elevated wire grid. Calibrated von Frey monofilaments were pressed perpendicularly against the central plantar aspect of the affected hindpaw until a slight buckling of the hair was observed. The 50% paw withdrawal threshold (PWT) was calculated by applying the classic Dixon’s “up-and-down” statistical paradigm [30].

### 4.5. Acetone Test

Cold hypersensitivity was evaluated using the acetone evaporation technique. A blunted 25-gauge syringe was used to carefully deposit a 0.3 mL drop of acetone onto the plantar skin of the hindpaw, creating a localized cooling sensation upon rapid evaporation. Aversive reactions, including rapid lifting, flinching, shaking, or licking of the stimulated paw, were monitored. This procedure was repeated for five independent trials per animal, with an inter-trial resting period of several minutes to prevent sensitization [31].

### 4.6. Pin Test

Punctate mechanical hyperalgesia was determined utilizing the pinprick assay. The plantar surface was gently poked with a blunt-tipped needle at an intensity strictly below the tissue penetration threshold. We distinguished between normal, transient reflex withdrawals and hyperalgesic aversive behaviors (defined as prolonged lifting, shaking, or licking of the paw). Each hindpaw received 10 consecutive stimuli separated by at least 10 s. Results were expressed as the occurrence rate (%) of hyperalgesic responses across the 10 trials.

### 4.7. Cell Culture and Transfection

Primary dorsal root ganglion (DRG) neurons were acutely dissociated from 4- to 6-week-old SD rats. Following isoflurane anesthesia and humane sacrifice, approximately 20–25 ganglia were bilaterally excised from the intervertebral foramina. The collected tissues were subjected to enzymatic digestion using a mixture of type I collagenase and neutral protease. This incubation was performed on an orbital shaker at 37 °C for 40 min. Post-digestion, the cell suspension was centrifuged (800 rpm, 3 min, 27 °C) to remove the supernatant. The resulting cell pellet was gently triturated and resuspended in a complete culture medium comprising DMEM supplemented with 10% fetal bovine serum and 1% penicillin/streptomycin. Finally, the dissociated neurons were seeded onto poly-D-lysine-coated (50 μg/mL) glass coverslips and maintained in vitro for up to 48 h prior to recording.

Human embryonic kidney 293 (HEK293) cells, provided by the Shanghai Institute of Biological Sciences (CAS, Shanghai, China), were propagated in DMEM containing 10% FBS and 1% antibiotics under standard conditions (37 °C, 5% CO2). For exogenous expression assays, a sequence encoding mouse Nav1.7 was custom-synthesized (GenScript) and subcloned into the pcDNA3.1(+)-C-DYK vector using BamHI and EcoRI restriction sites. This construct places Nav1.7 under the control of a CMV promoter with a C-terminal Flag tag. Using the JetPrime reagent, HEK293 cells were transiently co-transfected with the Nav1.7-Flag construct and a GFP reporter plasmid (Shenzhen Yanming Biotechnology) (Shenzhen, China). Transfection complexes were removed after 6–8 h of incubation, and the cells were replenished with fresh medium and treated with the designated compounds overnight before electrophysiological assessments.

### 4.8. Whole-Cell Patch Clamp Electrophysiology

Whole-cell patch-clamp recordings were performed on both native primary DRG neurons and transfected HEK293 cells, adapting established biophysical protocols [32]. The intracellular pipette solution contained (in mM): 140 CsF, 10 NaCl, 1.1 Cs-EGTA, and 15 HEPES (pH adjusted to 7.3 with CsOH; osmolarity 290–310 mOsm/L). The extracellular bath solution consisted of (in mM): 140 NaCl, 30 TEA-Cl, 10 D-glucose, 3 KCl, 1 CaCl2, 0.5 CdCl2, 1 MgCl2, and 10 HEPES (pH adjusted to 7.3 with NaOH; osmolarity 310–315 mOsm/L). Recording pipettes were fabricated from borosilicate glass and exhibited initial resistances of 2–4 MΩ when filled with the internal solution. All signals were amplified utilizing a HEKA EPC-10 USB amplifier, digitized via Patchmaster software (version 1.7), and subsequently analyzed with Fitmaster (version 2x92, HEKA Electronics, Lambrecht, Germany). Series resistance was compensated by 60–70%, and fast capacitive transients were systematically cancelled.

To evaluate the current–voltage (I-V) relationship, DRG neurons and HEK293 cells were clamped at holding potentials of −60 mV and −120 mV, respectively. Sodium currents were elicited by a series of 200-ms depolarizing voltage steps from −70 mV to +60 mV in 5-mV increments. Peak current amplitudes were normalized to whole-cell capacitance to determine the current density (pA/pF). For steady-state inactivation profiling, DRG neurons held at −60 mV were subjected to 1-s conditioning prepulses ranging from −120 mV to 0 mV (10-mV steps) to drive a fraction of the channels into the fast-inactivated state, followed immediately by a 200-ms test pulse to 0 mV to assess the remaining available channels.

For activation kinetics, the sodium conductance (G) was calculated using the equation G = I/(V-Vrev), where I is the peak current, V is the test potential, and Vrev is the reversal potential. The steady-state activation and inactivation data were fitted to standard Boltzmann equations to determine the half-maximal voltage (V_1/2_) and slope factor *k* using Fitmaster software and GraphPad Prism v10.

### 4.9. In Silico Molecular Docking

To ensure methodological transparency in accordance with established computational standards [33], molecular docking was performed using the Schrödinger Suite (version 2023-4, Schrödinger, LLC, New York, NY, USA). The 3D geometry of Quercetin (Que) was generated using the LigPrep module. Protonation states were assigned using Epik at a physiological pH of 7.4 ± 0.2, followed by energy minimization using the OPLS3e/OPLS4 force field. The crystal structure of the human Nav1.7 channel (PDB: 5EK0) was imported and refined using the Protein Preparation Wizard, which included the addition of missing hydrogens, optimization of hydrogen bond networks, and restrained minimization. The SiteMap module was used to validate the VSD4 extracellular pocket as the primary grid center. Receptor Grid Generation was performed to enclose this binding site with default van der Waals scaling. Flexible ligand docking was executed in Extra Precision (XP) mode to generate multiple poses. Binding affinities were scored using the XP Gscore, and the relative binding stability of the top-ranked conformation was further evaluated using the molecular mechanics generalized Born surface area (MM-GBSA) method.

### 4.10. Statistical Analysis

The Shapiro–Wilk test was performed to determine if the data were normally distributed, while the Grubbs’ test indicated no outliers in all datasets. Data from behavioral time-course assessments (Time × Treatment) were analyzed using a Two-Way Repeated Measures Analysis of Variance (ANOVA), followed by a Bonferroni’s multiple comparison post hoc test. The peak NaV current densities and the half-maximal voltages (V_1/2_) derived from the Boltzmann fits for activation and inactivation curves were analyzed using an unpaired two-tailed Student’s *t*-test (or Welch’s *t*-test when appropriate). Nonparametric data were analyzed using the Mann–Whitney test. All analyses were performed using GraphPad Prism v10. Comprehensive statistical methods, comparisons, and results (including F-statistics, *p* values, and 95% confidence intervals) can be found in Table 1. No data were missing, and results are presented as mean ± standard error of the mean (SEM). A significance level of *p* < 0.05 was considered statistically significant.

## 5. Conclusions

In conclusion, our findings establish quercetin as a specific functional modulator of Nav1.7 channels, highlighting its strong translational potential as a non-opioid therapeutic candidate for complex regional pain syndrome and related neuropathic pain conditions.

## Data Availability

The datasets generated and/or analyzed during the current study are available from the corresponding author on reasonable request.
